# Remarks on Rupture of the Uterus, with Report of a Case—Laparatomy—Recovery

**Published:** 1889-01

**Authors:** C. D. Josephson, L. G. Sellstedt

**Affiliations:** Stockholm


### Translations.

### *REMARKS ON RUPTURE OF THE UTERUS, WITH REPORT OF A CASE—LAPARATOMY—RECOVERY.*

By Dr. C. D. JOSEPHSON, Stockholm.

Translated from the original Swedish by Mr. L. G. Sellstedt.

[From *Hygeia*, September, 1888.]

Rupture of the uterus during parturition is of rare occurrence. Authors vary in regard to the frequency of this complication, and even the reports of the percentage of deaths taken as a whole, and with different methods of treatment, differ. This condition is easily explained. As to frequency, the occurrence of the most common causes become conclusive. Narrowness of the pelvis, neglected attention to cross position, and hydrocephalus fetus are the three most usual causes. Contraction of the pelvis, doubtless, occurs differently in different nations, but in a high degree it very rarely happens in our country. As the intelligence of midwives and even physicians increases, and access to the doctor becomes easier, ruptures of the uterus, caused by fault of position, ought to become rarer. This, too, should affect the frequency of such ruptures as are caused by hydrocephalus fetus when a timely puncture or perforation will protect the woman from the rupture of the uterus. Other malformations of the fetus, tumors in the uterus or pelvis, possibly the use of instruments without motive, perhaps a pendulous abdomen of the woman, irregular contractions of the muscular part of the uterus, seldom produce rupture, and ought in their actions to be neutralized by a proper prophylactic management, together with a careful carrying out of the treatment indicated. We may with safety assert that prophylaxis, such as is indicated and grounded chiefly on the epoch making work of Bandl,[1] has been of greater service in behalf of uterine ruptures than the treatment introduced after the rupture has taken place.

Rupture of the uterus occurs, according to Harris,[2] about once in 4,000 deliveries, while, according to the statement in Bandl (a. st.), Jolly gives one in 3,403, Ramsbotham one in 3,225. Bandl holds the occurrence much more common, and with his figures one in 1,183

(1) *Ueber Rupture d. Gebärmutter und ihre Medigen*, Vienna, 1875.

(2) *American Journal of Obstetrics*, 1880, Oct., p. 802.

approaches Churchhill's one in 1,318. The figures which give lesser frequency and which rest on larger numbers, and not alone from lying-in hospitals, but even and above all on private deliverances, seem more reliable, and even Harris's figures, one in 4,000, is perhaps too high.

How many of these women die, and how many are saved?

While Hugenberger gives ninety-five per cent., Braun eighty-nine per cent., and Jolly[1] eighty-two and six-tenths per cent. dead, Ames has found sixty-seven per cent.,[2] and Harris regards the mortality greater than is accepted by Jolly. These figures, taken from the time anterior to antiseptics, convey testimony of small value. That the percentage of mortality is very high even now, is certain. That manipulation after the rupture is often blind, and the indications for the different modes of treatment are still uncertain, we easily find by investigating the new literature on the subject. The result is likely, in the first place, to be decided by the manner in which the delivery has been managed before the occurrence of the rupture. If full asepsis has been observed, and no germs of infection are already found in the genitals of the woman, the prospects are not altogether bad. I naturally except the cases when death immediately intervenes in consequence of bleeding, or the shock; and when no treatment is possible.

When we speak of rupture of the uterus we ought to exclude the breaks in the portio vaginalis, which accompany every delivery, and in accord with the admitted phraseology, only regard the ruptures in the supra-vaginal cervix and corpus uteri. But we ought also to fully clear up the immense difference in the danger and the indicated method of treatment of a complete and incomplete rupture; a rupture that penetrates the entire wall of the uterus, and opens communication with the abdominal cavity, and a rupture that is arrested on its way, and leaves the peritoneum unhurt, and, therefore, does not open the cavity of the abdomen.

This difference has not been noticed in the older statistics, the reliability of which may, therefore, be further questioned. Every one may judge of the significance of this in regard to management. The imperfect rupture ought almost never to be treated but in one way, viz., by extraction of the fetus *per vias naturales*, and draining of

(1) Lusk: "The Science and Art of Midwifery," London, 1884, p. 565.

(2) *American Journal of Obstetrics*, 1881, p. 361.

the cavity with the drainage-tube or, especially when the bleeding is considerable, with iodoform gauze, and more or less tamponing. There can seldom be question of laparatomy, unless the hemorrhage is so great as to call for sutures.[1]

The prognosis for an incomplete rupture is quite different. Although even here the lesion may be so great, the external bleeding or the sub-peritoneal hematoma so considerable, that death follows in a short time. But with proper management the woman's prospect ought to be fairly good.

Quite otherwise with the complete rupture.

If the fetus is still fully, or in greatest part, within the genital canal, or, as often has happened, the rupture has not been discovered till the fetus has been extracted, the general judgment would be, that we ought to be satisfied with the removal of the fetus and the placenta in the best way possible, and that laparatomy ought not to be resorted to.

Shall we drain through the rupture?

To this question answers differ. While most of the German authors, especially the schools of Berlin, regard drainage useful, some have[2] expressed themselves against it as hurtful and, at the best, useless. It has been held that, through the motions of the patient, and especially with irrigation through the drainage-tube, secretions and air may be driven into the cavity of the abdomen, and produce infection, and that irrigation can hinder the adhesion of the surfaces of the peritoneum, which otherwise, in a short time, would shut out the abdominal cavity from the outer world. In operative gynecology, the drainage-tube is already almost abandoned. Why should it be necessary here, if no sepsis was found when the rupture was discovered; and if infection is found, of what use is the drainage? It is shown that within a short time, most likely within the first forty-eight hours, the drainage-tube is surrounded by a new-formed tissue of adhering surfaces of the peritoneum, which render the drainage of the cavity illusory. If seeds of infection are found in the abdominal

(1) Halbertsman (*Nederl. Tijdschr. v. Geneerk*, 1880, N. 36) performed laparatomy and extirpation of the uterus after an incomplete rupture caused during turning in pelvical contraction (c. v. 8. cm.) and large fetus (4,590 gm.), but the operation was performed as much in the interest of the fetus, which was saved. Even the mother got well.

(2) Kronner: Centralbl. f. Gyn., 1884, No. 26, p. 369. Kaltenbach: Arch. f. Gyn., Bd. 22, p. 123. Felsenreich: Arch. f. Gyn., Bd. 17, p. 490.

cavity, drainage, even within the first twenty-four hours, has no power to remove the secretions from all the corners between the intestines, and the natural drainage, caused by the pressure in the abdominal cavity, assisted by compressor bandages and the layers of weight, which drive the secretions down towards the rupture, aided also by the position of the patient, the upper part of whose body is raised by the bedclothes, seem to be able to produce the same effects as the drainage-tube. When to this is added that the place of the rupture—usually in the back or side wall of the cervix—is convenient for the outflow, it appears that many things would go to show that the drainage-tube may be dispensed with. Perhaps, however, drainage with iodoform gauze may be proper, and it is very likely that it is not subject to the same inconveniences that are attributed to the drainage-tube.

Statistics leave no reliable answer to this question. I have certainly, in the literature of these last fifteen years, sought to gather responsive reports. I have found, of twenty cases treated without, and of eleven with, drainage, ten to nine respectively recovered. These figures speak in favor of drainage, though they are altogether too small to prove anything.

If the fetus has wholly, or in greatest part, pushed out into the abdominal cavity, the question of laparatomy becomes pertinent. Even on this point opinions are greatly divided. While Harris and most American authors look on laparatomy as giving the best prospects, the German authors hold that extraction, *per vias naturales*, with or without subsequent drainage, affords greater advantages. Statistics do not reply to the question. While Trask[1] found seventy-six per cent. saved by laparatomy, Harris found fifty-two per cent., and I myself have, in twenty-four cases, including my own, found only six saved, thus twenty-five per cent. While all these cases have been published during the last fifteen years, it is to be feared that many with fatal termination remain unpublished, and that even twenty-five per cent. is too high. Schroeder[2] regards laparatomy as almost without prospect. He has lost all his seven ruptures of the uterus treated with laparatomy, but, on the other hand, has seen five out of seven saved that were treated with drainage. Schauta[3] has

(1) Harris, a. st.

(2) *Lehrbuch d. Geburtshülfe*, 7 *Auff.*, Bonn., 1882, p. 673.

(3) *Operation Geburtshülfe*, Wien., 1885, p. 222.

found that, of eighty-eight cases of ruptnre of the uterus, forty-nine were saved by laparatomy, thus 55.68 per cent., and considers laparatomy indicated; so Barnes,[1] who goes still further, and seems to order laparatomy, followed by extirpation of the uterus. This last operation seems, however, to give the poorest prospects, since of ten cases described in medical literature, only one has resulted in health, that of Halbertsman, and in that often-mentioned case the rupture was incomplete, and the conditions, besides, uncommonly favorable. Besides, when even Porro's operation, in later times, has had to give way for the conservative Cesarean section, even this seems to have no future. In two cases of rupture during the latter part of pregnancy, it has nevertheless been tried with success.[2]

It is, therefore, difficult to give certain indications for the treatment, but it seems as if a complete protrusion of the fetus into the cavity of the abdomen, with its filth and blood, secretions and waters, would be regarded as indicating laparatomy, that we might as much as possible clean the abdominal cavity, and this particularly if the delivery has taken place some time before the rupture, so that the secretions are found to have commenced to decompose. It is certainly true that we cannot count on a *complete* peritoneal toilet, but the greatest part of the secretions, and nearly all the blood, can be taken up, by which we deprive the bacteria of an excellent element of nutrition.

In three conditions, laparatomy seems to be the only proper method:

*First*—If copious bleeding sets in, which in no other way can be stayed, we ought to resort to the abdominal section, and by ligature of the bleeding vessels or suture of the rupture, stop the hemorrhage. But such cases only give hope of success when the outward circumstances permit its immediate practice. Thus only in obstetrical institutions, and there it is probable that uterine ruptures nowadays have become extremely rare since prophylaxis through Bandl has assumed full sway.

*Second*—If the rupture is already contracted so that the fetus only with great difficulty, or not at all, can be brought out through the break, laparatomy must be resorted to. It would be much more dangerous to attempt extraction *per vias naturales* under such circum-

(1) *Obstetric Med. and Surg.*, London, 1885, Part II., p. 347.

(2) *Slavjansky Centrbl. f. Gyn.*, 1886, No. 14, p. 222. *Plenis Centrbl. f. Gyn.*, 1885, No. 47, p. 737.

stances; we should still more utterly insult the uterus and call forth hemorrhage. The same holds good if the fetus is removed, but the placenta has remained in the abdominal cavity when the rupture no longer permits the insertion of the hand. The fetus may also lie so bedded in with the intestines and the omentum that we may be induced to resort to laparatomy for fear of, by a difficult extraction, injuring these.

*Third*—If the uterus is already ruptured before the membranes have broken, and the whole ovum is carried into the abdominal cavity, it is better to make laparatomy and take out the ovum without soiling the cavity of the abdomen with the liquor amnii than to burst the membranes after the hand, if such thing is possible, has been carried through the opening, and to extract through it. These cases probably give good indications for laparatomy.

Besides these, when the indications for laparatomy are plain, many cases exist when the choice of treatment may depend on the observation of outward circumstances. I will here remark that the woman's condition may at the first examination be so poor, that we have the right to wait till her powers are somewhat raised. But to adopt the so-called expectant method and leave the fetus in the abdominal cavity in hope that it will be dismembered, at last to be eliminated through nature's own assistance, would probably no longer occur to any one, although even in such cases health has been restored.

Lastly, there remains a question to answer. How shall the rupture of the uterus be treated in laparatomy?

Shall it be stitched together or left open? I do not believe that in general it is wise to sew it up completely. If the uterus is well contracted, the rent usually is not greater than is needed for the outflow of the secretions found in the cavity of the abdomen. It is remarkable how quickly even very large ruptures, which leave only a small part of the cervix undivided, become smaller under the involution of the uterus, and how completely they grow together without other marks than a slight cicatrix. To sew them together would be to deprive one's self of the advantage of a safe result, so much more necessary as no great hopes are entertained of a full aseptic result. It is usual after colpo-hysterectomy to leave the bottom of the vagina completely or in part open, and practice has shown its advantage. Why sew together the ruptures which, after a couple of days, are rather less than greater, than this opening? If the uterus is very loose and the rupture very large—above all, if bleeding from its edges takes place—it ought to be stitched together wholly or in part. In regard to drainage, I have expressed myself above.

Finally, I will describe a rupture of the uterus treated by myself:

Mrs. Charlotta Sillén, thirty-eight years old, has heretofore had five living children. All the labors normal, though long. The first was eleven years since, and the last two years ago. Has never lain sick, but during her last pregnancy has been poorly, and has had a considerably pendulous abdomen. I was called in the night of the 26th of March, this year, to the woman, who lived at Djursholm, about a mile[1] from Stockholm. On my arrival, I found her sitting bolstered up high in the bed, complaining of pains in the abdomen. The pulse was small and quick—120 a minute—and she impressed me as being very low-spirited, and threatened with collapse. I suspected a rupture of the uterus, and tried to get some information from the midwife. She, old and shaky, was not very clear, and insisted that she had felt the head coming forwards. The pains had began the 25th, three o'clock A. M. The waters had passed off at noon, after which the pains had been much stronger and intense, and between five and six they had *gradually* ceased. After this, no pains; the woman had lain in half-slumber, had not felt any violent sudden pain; neither had the pains ceased at once. No considerable outward bleeding had occurred, but she had herself felt how the fetus, little by little, had shifted itself higher and higher up into the abdomen. The midwife had comforted her with the assurance that the pains would doubtless return, and only late in the evening they concluded to call a physician. External examination, with the hand, showed that the abdomen was extremely tender; the head of the fetus was felt under the skin high up in the neighborhood of the liver, and the small parts over the middle and left side. At the left, in the iliac region, a larger resistance was felt, which afterwards proved to be the uterus. No fetal sounds were heard.

The vagina was carefully rinsed out with a solution of sublimate, and the woman was chloroformed.

The vagina was wide; contained some blood; high up was felt an irregular, soft substance, placenta, and to the left the uterus, contracted and allowing the insertion of three fingers into the cavity. The placenta was removed, the navel-string cut, and I could now carry the hand through the break, which was filled with placenta, up into the cavity of the abdomen, where I felt the head of the fetus. The pelvis and the feet lay up against the epigastrum, surrounded by the small intestines and omentum, and were out of reach. I felt, with the outer hand, the fingers of the inner hand close to the wall of the abdomen. Out after my arm flowed a considerable mass of dark, thick blood. The condition of the woman became worse and worse; the pulse small. I felt the aorta with the inner hand as a weakly pulsating string, and as I believed the woman was dying, refrained from an extraction, which possibly might have injured the intestines and omentum, and certainly still more have torn open the rupture, brought about hemorrhage, and perhaps hastened death. The chloroform was discontinued, and the woman awakened slowly, lay there with a stertorous respiration, with staring eyes and little pulse, and to all appearance dying. I

(1) A Swedish mile is nearly equal to seven English. Tr.

explained to the husband that she did not have many hours to live, and prescribed a solution of morphine, 1.25 c. g., every other hour, and went home. This was four A. M. About nine o'clock a message came that she still lived, was sensible, and spoke. I waited for information till noon, in order to drive out and perform laparatomy, but got none, and then concluded that she was dead. But the next morning I was informed that she still lived, had slept during the night, and seemed better. I then determined on laparatomy, poor as the prospects were, drove out, had her removed from the peasant hut, where she lived, up into a room at the inspector's of the place, and undertook laparatomy the 27th March, one P. M., forty-four hours after the occurrence of the rupture. The woman was now very weak, but the temperature in the axilla only 37.9 C., the pulse 108, color of the skin somewhat icterish. The rupture had contracted, allowing scarcely the finger to enter. It stretched upward and to the right, in the posterior wall of the cervix. The uterus was contracted, the lochia without offensive smell. The bladder was tense, and was tapped.

I was assisted by Dr. L. Remahl, and the chloroform was attended to by Fr. Gustafson.

*Operation.*—Abdominal incision below the navel 15 c. m. downward; almost no bleeding; thin abdominal walls. The fetus lay as before, with the head to the right, the back and left side forwards; was easily extracted by the left leg. The intestines were ingested, but no particular meteorism, no exudation, no adhesions, were visible. A considerable quantity of blood had run out by the cut through the peritoneum, and the rest, about one or two litres, was baled out with the hands and taken up with cotton points, wrung out in a solution of boracic acid; the lesser pelvis contained a quantity of blood which was taken up in the same manner. The uterus lay in the median line, contracted and of the usual size at this stage of parturition. The rupture was seen and felt in the posterior cervical wall from the bottom of the fossa Douglasa upward, and to the right; in fact, somewhat above the os internum, but lay with its lines in apposition, and were not sewn together. No drainage tubes were inserted. The intestines were cleaned as well as could be done with cotton points, but were not eventered. The wound in the abdomen was closed with deep and superficial sutures and separate peritoneal suture. The operation lasted forty minutes. Bandage of iodoform and boracic acid gauze compresses; the nearest wrung out in a solution of sublimate. Cotton and body bandage. The vagina was cleaned, and into the cervix was inserted a strip of iodoform gauze, the higher part of which was pressed into the rupture. The patient had borne the chloroform and operation well; true, the pulse had gone up to 120, but went down to 108 afterwards. Patient soon awoke and was sensible and free from pain.

The child was large, but not unusual; it was neither weighed nor measured. No sign of fetal swelling, which could indicate the position, was discovered. Patient had morphine 1.5 c.gm. three times a day; in the evening, the temperature 38.4 C. (in axilla unless otherwise stated).

*Second Day.*—Stitch in the left side; nausea; has slept in the night; makes water herself; pulse, 95; temperature, 37.40° C. in the rectum; champagne, soda water and iced milk.

*Third Day.*—Considerable meteorism, tenderness in the right side of abdomen; pulse, 96; temperature, 38.4°; at twelve, 39° C. (in the rectum); iodoform strips in the cervix changed; a few remains of membrane removed, and a two per cent. carbolic wash in the cervix; enema, twelve and four P. M., remains; temperature in the evening, 38.1°.

*Fourth Day.*—Slept tolerably; enema comes back with some wind; continued meteorism; vomiting; bandage removed, and is substituted by a compress of iodoform gauze, fastened with broad strips of sticking-plaster; two large bladders of ice on the abdomen; flowing from the vagina has bad odor; carbolic wash; temperature, 38.4° to 39° (in rectum).

*Fifth Day.*—Condition better; meteroism less; enema, *wind;* nausea; black coffee; temperature, 38.3° C–38.3°.

*Sixth Day.*—Slept; great movement after enema; no pain in the abdomen; carbolic wash daily; temperature, 38.2°-38° C.

*Seventh Day.*—Meteroism lessened; much wind; tenderness over the right lower part of the abdomen, where a slight resistance is felt; mild nausea; eats milk and eggs; the flowing offensive; sublimate wash in cervix; temperature, 38°–38.1° C.

*Eighth Day.*—37.5°–38.4° C.

*Ninth Day.*—37.7°–38.1° (rectum); pulse, 100; to-day, half-loose movements; no gripes; urine of normal color. The sutures were taken out, except two deep ones and two superficial. Abdominal wound healed p. p. Uterus, which reaches up to three fingers below the navel, is adherent to the lower part of the cicatrix of the abdomen. In the right parametrium, a resistance; cervix open for a finger; condition good; ice bladders are removed. Patient has eggs, beef and milk soups.

*Tenth Day.*—Six movements; temperature, 37.5°–38° C.

*Eleventh Day.*—Six movements, 37.3°–38.3° C. (rectum) half a teaspoonful of Rosen's breast-drops; the remaining sutures removed.

*Twelfth Day.*—37°–37.5° C.; no movement.

*Thirteenth Day.*—37°–37.4° C.; a movement.

*Fourteenth Day.*—The patient was brought in to the Seraphim's Hospital, where Prof. Salin had the goodness to receive her in his department. The abdomen is soft, not tender, over the right parametrium; uterus adherent to the lower abdominal scar, and movable with the wall of the abdomen; cervix somewhat fixed posteriorly, and to the right and in the right parametrium, slight resistance is felt. The finger can be carried into the cervix, and feel there, between the posterior and right wall, a deep vertical groove (the cicatrice after the rupture).

Warm washes, wet warm compresses. After this the patient was free from fever. The resistance was wholly gone after a week. The twenty-ninth day after the operation she was permitted to leave her bed, and was discharged forty days after the operation. This day, on investigation, no resistance was felt, but a string-formed thickening in the right parametrium, proceeding from the cervix. Uterus continues adherent to the cicatrix of the abdomen, and the cervix open; the groove in the cervix palpable; the sound could not be carried in from it into the parametrium.

The measures of the pelvis gave, sp. il., 23 c. m.; cr. il., 27 c. m.; c. e., 18 c. m. The inner measures seem normal, and the promontorium cannot be reached.

What the cause of this rupture of the uterus was is not possible with certainty to determine. It is possible that a neglected cross-position had existed. The statement of the midwife that she had felt the head of the fetus, is without significance; she appeared anything but reliable, and, besides, knew nothing of parturition. If we believe the position to have been normal, the pendulous abdomen may account for the accident, in that the uterus had got a disadvantageous position against the axis of the pelvis; and the head of the fetus, instead of being driven out in the direction of the axis of the pelvis, had been driven backwards against the cervical wall, which at last gave way. Against the management may be objected that extraction was not immediately resorted to. The reasons for this are given in the description of the case, and I will further point out that when the condition still, ten hours after the occurrence of the rupture, when the first shock ought to have been overcome, was so poor, the prospects were more than miserable. That the woman's strength had been underrated, the result showed, and it may serve as a lesson in similar cases. It is possible that the result had been equally good if extraction had been resorted to at once. That the operation was undertaken so late depended on outward circumstances already explained.

